# Genome-wide DNA methylation profiles in progression to *in situ* and invasive carcinoma of the breast with impact on gene transcription and prognosis

**DOI:** 10.1186/s13059-014-0435-x

**Published:** 2014-08-22

**Authors:** Thomas Fleischer, Arnoldo Frigessi, Kevin C Johnson, Hege Edvardsen, Nizar Touleimat, Jovana Klajic, Margit LH Riis, Vilde D Haakensen, Fredrik Wärnberg, Bjørn Naume, Åslaug Helland, Anne-Lise Børresen-Dale, Jörg Tost, Brock C Christensen, Vessela N Kristensen

**Affiliations:** Department of Genetics, Institute for Cancer Research, OUS Radiumhospitalet, Montebello, 0310 Oslo, Norway; The K.G. Jebsen Center for Breast Cancer Research, Institute for Clinical Medicine, Faculty of Medicine, University of Oslo, 0318 Oslo, Norway; Oslo Centre for Biostatistics and Epidemiology, Department of Biostatistics, University of Oslo and Research Support Services, Oslo University Hospital, 0424 Oslo, Norway; Department of Community and Family Medicine, Section of Biostatistics and Epidemiology, Geisel School of Medicine at Dartmouth, Hanover, NH 03755-1404 USA; Department of Pharmacology and Toxicology, Geisel School of Medicine at Dartmouth, Hanover, NH 03755-1404 USA; Institut de Génomique, Laboratory for Epigenetics and Environment, Centre National de Génotypage, CEA, 91000 Evry, France; Department of Clinical Molecular Biology and Laboratory Science (EpiGen), Division of Medicine, Akershus University hospital, 1476 Lørenskog, Norway; Department of Surgery, Akershus University Hospital, 1478 Lørenskog, Norway; Department of Breast and Endocrine Surgery, Oslo University Hospital, Ullevål, 0450 Oslo, Norway; Department of Surgery, Uppsala Academic Hospital, Uppsala University, Uppsala, SE-75185 Sweden; Department of Oncology, Oslo University Hospital Radiumhospitalet, 0379 Oslo, Norway

## Abstract

**Background:**

Ductal carcinoma *in situ* (DCIS) of the breast is a precursor of invasive breast carcinoma. DNA methylation alterations are thought to be an early event in progression of cancer, and may prove valuable as a tool in clinical decision making and for understanding neoplastic development.

**Results:**

We generate genome-wide DNA methylation profiles of 285 breast tissue samples representing progression of cancer, and validate methylation changes between normal and DCIS in an independent dataset of 15 normal and 40 DCIS samples. We also validate a prognostic signature on 583 breast cancer samples from The Cancer Genome Atlas. Our analysis reveals that DNA methylation profiles of DCIS are radically altered compared to normal breast tissue, involving more than 5,000 genes. Changes between DCIS and invasive breast carcinoma involve around 1,000 genes. In tumors, DNA methylation is associated with gene expression of almost 3,000 genes, including both negative and positive correlations. A prognostic signature based on methylation level of 18 CpGs is associated with survival of breast cancer patients with invasive tumors, as well as with survival of patients with DCIS and mixed lesions of DCIS and invasive breast carcinoma.

**Conclusions:**

This work demonstrates that changes in the epigenome occur early in the neoplastic progression, provides evidence for the possible utilization of DNA methylation-based markers of progression in the clinic, and highlights the importance of epigenetic changes in carcinogenesis.

**Electronic supplementary material:**

The online version of this article (doi:10.1186/s13059-014-0435-x) contains supplementary material, which is available to authorized users.

## Background

Epigenetic marks (and DNA methylation in particular) are known to be deregulated in cancer. Cancer-specific changes include hypermethylation of CpGs in gene promoters [1], hypomethylation of non-CpG island CpGs [2], and overall increase in variation of methylation [3,4]. DNA methylation patterns are also associated with histopathological parameters such as hormone receptor status, *TP53* mutation status, histologic grade, stage, and survival time [5–9].

Ductal carcinoma *in situ* (DCIS) of the breast is a neoplasm where the cells are confined by the basement membrane of breast ducts. DCIS is a precursor of an invasive breast carcinoma (IBC). Treatment of DCIS consists of surgical excision in the form of either breast-conserving surgery (that is, wide local resection or sector resection) or removal of the entire breast parenchyma (mastectomy). Treatment by mastectomy results in very few recurrences but is considered over-treatment in a majority of cases. Approximately 30% of patients treated by breast-conserving surgery alone are reported to develop a local recurrence after 15 years follow-up and the risk of local recurrence is reduced by half if postoperative radiotherapy is given [10,11]. To avoid overtreatment of patients with DCIS it would be of great value to be able to predict which patients have potentially malignant and invasive tumors and are likely to experience recurrence of disease.

Epigenetic studies of breast tissue report aberrant methylation levels already present in benign neoplastic breast lesions such as columnar cell lesions and ductal hyperplasia [12,13]. More studies have reported aberrant methylation levels in DCIS (summarized in [11]). Most of these studies reported methylation levels of only one or a few genes, while two studies have used genome-wide approaches. These studies reported 108 and 214 CpG islands (CGIs), respectively, to be hypermethylated in DCIS compared with normal tissue and that these CGIs were enriched for homeobox genes [14,15].

Studies of benign or premalignant tumors from a variety of organs have revealed that some of these lesions have epigenetic characteristics that separate them both from normal tissue and from the malignant tumors. These studies include results from meningiomas of the brain [16,17], gastric lesions [18,19], cystadenomas of the ovary [20], colorectal lesions [21,22] and uterine leiomyoma [23,24]. Taken together, these data suggest that epigenetic changes occur early in cancer development and as such have great potential as biomarkers in addition to increase our biological understanding of progression of cancer.

In this study, we investigate methylation patterns in a total of 285 fresh frozen tissue samples, including 46 normal breast tissue samples from healthy women, 22 pure DCIS, 31 mixed DCIS-IBC and 186 IBC of stage I and II. Validation was performed using 583 breast cancer samples from The Cancer Genome Atlas (TCGA), as well as in an independent set of DCIS and adjacent normal tissues.

The aim of this study was to investigate DNA methylation patterns during progression of breast cancer. Genome-wide profiling allows identification of molecular changes that occur during neoplasia as well as changes that are required for a tumor to acquire invasive capabilities. Additionally, the association between methylation and survival of patients was studied, and a prognostic signature was identified. The correlation between methylation and expression was incorporated into the analyses. These findings could improve our understanding of the biological mechanisms that occur during progression of breast cancer, and contribute to identification of biomarkers for risk-related classification of patients with *in situ* and invasive breast cancer.

## Results

### Tumor classification based on DNA methylation

**Figure 1:**
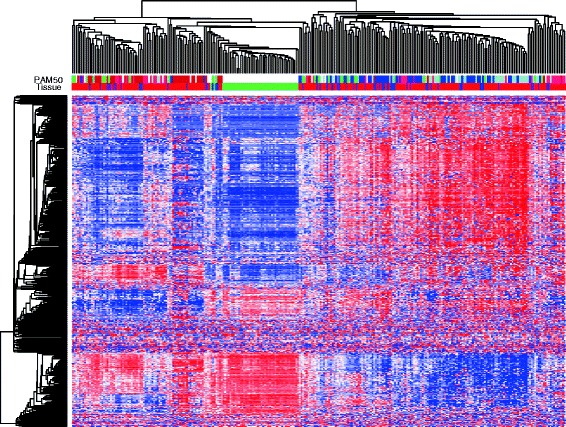
**Hierarchical clustering of the methylation level of the 500 most variable gene regions.** Tissue types (green, healthy breast; blue, DCIS; purple, mixed DCIS-IBC; red, IBC) and PAM50 subtype (dark blue, luminal A; light blue, luminal B; pink, HER2-enriched; red, basal-like; green, normal-like) are indicated.

Genome-wide DNA methylation analysis was performed on a total of 239 breast tumor samples and 46 normal samples. The tumor samples included 22 pure DCIS, 31 mixed DCIS-IBC and 186 pure IBC. A gene region collapsed data set was constructed to reduce the dimensions of the data and to study the methylation in the functional regions of the genes. Each gene is represented by up to six methylation values representing the respective functional regions as described in Materials and methods). Hierarchical clustering was performed to explore the structure of the DNA methylation data, using methylation levels of the 500 most variable gene regions (Figure 1). The samples were divided into two main clusters, where one contained all normal samples as well as tumor samples, and the other contained only tumor samples. Basal-like tumors were enriched in the cluster containing the normal samples while luminal A and luminal B tumors were mostly found in the other cluster. DCIS and mixed DCIS-IBC tumors were found in both clusters.

Differentially methylated gene regions were identified between the five intrinsic subtypes of breast cancer. For a locus to be considered differentially methylated, the minimum difference between the median methylation levels in the groups was 0.1 (10%) and the false discovery rate (FDR) q-value was smaller than 0.01 (1%); 16,723 gene regions were differentially methylated between the intrinsic subtypes of breast cancer (listed in Additional file 1). Hierarchical clustering of the invasive tumors using these regions confirmed that the basal-like and normal-like tumors showed clearly distinct profiles compared with the luminal-like tumors (Additional file 2).

### Correlation between DNA methylation and gene expression

Correlation between DNA methylation level and gene expression was investigated to assess to what degree gene expression may be influenced by DNA methylation in breast cancer. Gene expression level was tested for correlation to both methylation level of single CpGs within 100 kb of a transcription start site (TSS) and methylation level of gene regions.

**Figure 2:**
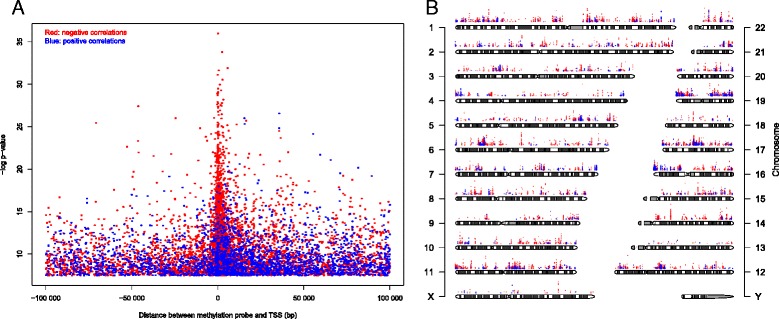
**CpGs whose methylation level significantly correlated with gene expression (Bonferroni corrected** ***P*** **-value <0.05). (A)** Significance level of correlation between methylation level and gene expression plotted against distance between the CpG and transcription start site (TSS). Red dots represent negative correlation and blue dots represent positive correlation. **(B)** Significance level and genome-wide distribution of correlation between methylation level and gene expression.

Pearson correlation was calculated between gene expression and methylation level of CpGs within 100 kb of a TSS, and an association was considered significant if the Bonferroni corrected *P*-value was smaller than 0.05. By this definition, the methylation level of 9,800 CpGs was significantly correlated with the expression of 2,960 genes (Additional file 3). The expression level of 2,558 genes was negatively correlated with the methylation level of at least one CpG, while the expression level of 852 genes was positively correlated with the methylation level of at least one CpG. The positive correlations were quite evenly distributed relative to TSSs (±100 kb; Figure 2A). The negative correlations were also found at all distances relative to TSSs (±100 kb; Figure 2A), but they were found to be enriched close to TSSs (1,000 bp upstream to 5,000 bp downstream). The CpGs that correlated with expression were distributed across the whole genome, and were enriched on chromosomes 1, 17 and 19 (Figure 2B).

**Figure 3:**
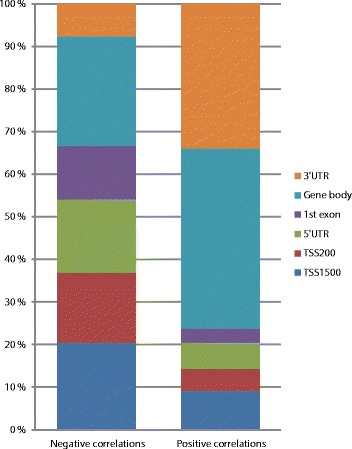
**Significant correlation (Bonferroni corrected** ***P*** **-value <0.05) between gene expression and methylation level of gene regions.** Bar plot showing the distribution of negative and positive correlations relative to the functional regions of genes. The distribution is notably different for the negative versus positive correlations.

Pearson correlation was also calculated between gene expression and the methylation level of each respective gene region. The expression of 1,719 genes significantly correlated with methylation level in at least one gene region (Additional file 4). The expression of 1,445 genes negatively correlated with the methylation level of at least one gene region, and the expression of 355 genes positively correlated with the methylation level of at least one gene region (Figure 3). Of the negative correlations between methylation and expression, almost 40% were found upstream of TSSs (TSS1500 and TSS200 subregions), while only about 15% of the positive correlations were found upstream of a TSS. The rest of the negative correlations were distributed in the 5’ UTR, first exon and gene body, while less than 10% of the negative correlations were found in the 3’ UTR. Of the positive correlations, 40% were found in the gene body and 30% were found in the 3’ UTR, meaning that more than 70% of positive correlations were found outside of promoter regions.

### Methylation changes during progression of breast cancer

To identify differentially methylated CpGs during progression of breast cancer, Significance Analysis of Microarrays (SAM) was applied to the complete methylation data set with all CpGs. For a locus to be considered differentially methylated, the difference between the median methylation levels in the two groups had to be at least 0.1 (10%) and the FDR q-value had to be smaller than 0.01 (1%). The differences in methylation levels between normal tissue and DCIS were substantial. A high degree of CpG methylation deregulation during neoplastic transformation may have important implications for a better understanding of breast cancer progression. Thus, to identify the most biologically relevant alterations we examined differences between normal tissue and DCIS in two independent patient cohorts. Only the significant CpGs or regions that were differentially methylated in both datasets are reported. Comparing normal tissue and DCIS revealed that 16,949 CpGs were differentially methylated, representing 5,659 genes. Comparing DCIS and IBC revealed that 2,000 CpGs were differentially methylated. These CpGs represented 1,076 genes, and 1,745 of the CpGs were hypermethylated while 255 of the CpGs were hypomethylated (Table 1). All differentially methylated CpGs are shown in Additional files 5 and 6.

**Table 1:** **Differential DNA methylation during progression of breast cancer**

	**Healthy-DCIS** ^**a**^	**DCIS-IDC**
Total differentially methylated CpGs^b^	16,949	2,000
Total represented genes	5,659	1,076
Total differentially methylated gene regions^c^	1,249	166
Total represented genes	1,011	154

^a^Analysis performed in two independent datasets. Only concordant results in both datasets are reported. ^b^All individual CpGs differentially methylated between normal and DCIS, and between DCIS and IBC. ^c^Gene regions differentially methylated between normal and DCIS, and between DCIS and IBC. Differential methylation was determined by SAM analysis (FDR q-value <1% and difference in median of groups >10%).

SAM was also applied to the gene region collapsed data, and comparing normal tissue and DCIS in the two independent patient cohorts revealed 1,249 differentially methylated gene regions representing 1,011 genes. In comparison, 166 gene regions representing 154 genes were differentially methylated between DCIS and IBC (Table 1). All differentially methylated gene regions are shown in Additional files 7 and 8.

To identify pathways for which the differentially methylated genes between normal tissue and DCIS were enriched Ingenuity Pathways Analysis was performed. This analysis was performed on the genes represented by differentially methylated gene regions rather than single CpGs. The differentially methylated genes between normal tissue and DCIS approached a significant threshold for an enrichment in the agranulocyte adhesion and diapedesis pathway (*P* = 0.053) and the granulocyte adhesion and diapedesis pathway (*P* = 0.084) (Table 2).

**Table 2:** **Ingenuity canonical pathways enriched for differentially methylated genes (represented by gene regions) between normal tissue and DCIS**

**Ingenuity canonical pathways**	**Benjamini-Hochberg corrected** ***P*** **-value**	**Ratio**
Agranulocyte adhesion and diapedesis	0.05	21/175
Granulocyte adhesion and diapedesis	0.08	19/165

The methylation level of four genes (*CPA1*, *CUL7*, *LRRTM2* and *POU2AF1*) increased both from normal to DCIS and from DCIS to IBC, while 10 genes (*ARSJ*, *CES8*, *FAIM2*, *GPRC5B*, *ICAM2*, *P4HA3*, *PGLYRP2*, *PLOD1*, *PNMAL2*, *STAP2*) showed a decrease in methylation between normal and DCIS, but an increase in methylation from DCIS to IBC.

### Survival analysis

**Figure 4:**
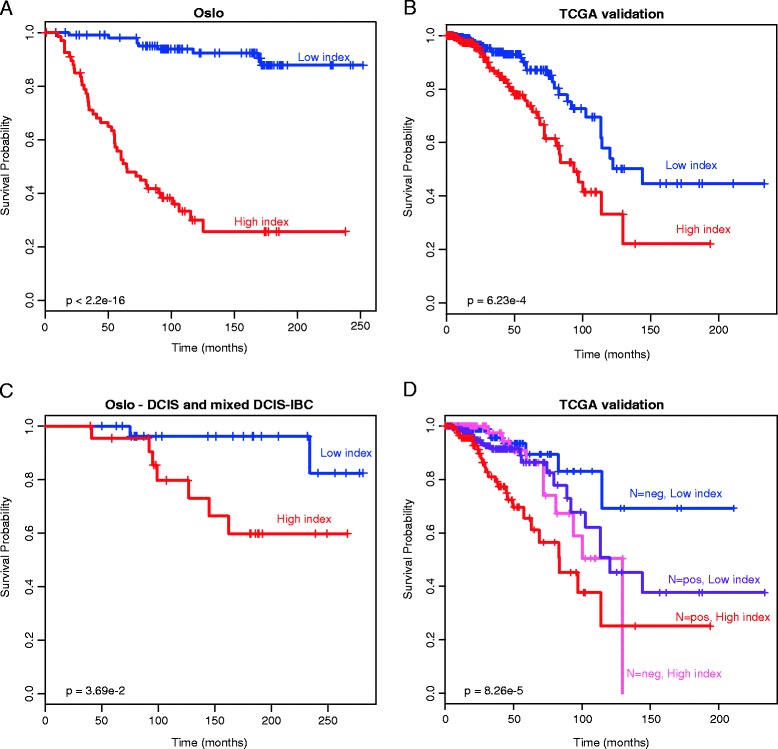
**Application of the DNA methylation-based prognostic signature for patients. (A)** In the original data (n = 176); **(B)** in the TCGA validation (n = 583); **(C)** with either DCIS or mixed DCIS-IBC (n = 52). **(D)** Classification with the DNA methylation-based prognostic signature was complementary to classification by lymph node status.

To identify CpGs for which methylation level may predict survival, Lasso regularization was performed. The analysis was performed using methylation level of single CpGs, preselected to be correlated with gene expression. A survival signature that consisted of 18 CpGs was identified (Table 3). The methylation level of these CpGs correlated with the expression level of 26 genes, including *IRF6*, *TBX5*, *CSNK1G2*, *MACF1*, *KCTD21* and *EPN3* (Table 4). Of the genes associated with the signature, 15 negatively correlated with methylation level and 11 positively correlated with methylation level. No canonical pathways were found significantly enriched among the 26 genes. Of the genes in the signature, 17 were differentially methylated between normal and DCIS, and 4 were differentially methylated between DCIS and IBC. The survival signature was applied to patients with invasive tumors (n = 176) as explained in Materials and methods. The patients segregated exceptionally well into high- and low-risk groups according to breast cancer-specific survival (hazards ratio (HR) = 13.7, *P* < 2.2e-16; Figure 4A).

**Table 3:** **DNA methylation-based prognostic signature identified by Lasso**

**Probe**	**Coefficient**
cg05809947	−0.10
cg12219311	−0.08
cg26466505	−0.07
cg20691428	−0.07
cg20869305	0.09
cg22174844	0.16
cg26225829	−0.13
cg04947065	0.06
cg16575694	−0.09
cg16559598	0.07
cg08729004	−0.09
cg13635578	−0.03
cg13744452	−0.04
cg04817034	−0.02
cg07130508	0.02
cg00226265	−0.04
cg13749939	0.06
cg25817165	0.05

A positive coefficient reflects that a high methylation level is associated with adverse prognosis. These coefficients are used to classify patients into high and low risk groups.

**Table 4:** **Genes whose expression level correlated with methylation level of CpGs in the survival signature**

**Gene**	**Probe**	**Gene region**	***P*** **-value (methylation-expression)**	**Coefficient (methylation-expression)**
*ACAP3*	cg16575694	Other	8.5E-03	−0.5
*ALG8*	cg04817034	Other	4.8E-02	−2.7
*BLZF1*	cg08729004	Other	2.6E-02	0.9
*C1orf74*	cg20869305	Other	5.9E-04	38.7
*CBS*	cg05809947	Other	5.8E-03	−4.5
*CEND1*	cg13749939	Other	3.5E-04	1.2
*CNDP2*	cg25817165	Body	1.2E-05	−1.0
*COLEC11*	cg04947065	Other	8.7E-03	28.9
*CSNK1G2*	cg12219311	Other	1.5E-02	−0.6
*DIEXF*	cg20869305	Other	4.5E-04	32.8
*EPN3*	cg13635578	Other	3.7E-02	−1.6
*FCGRT*	cg26225829	Other	3.6E-05	2.8
*FKBPL*	cg00226265	Other	2.7E-02	−1.7
*IRF6*	cg20869305	Other	1.4E-03	61.1
*KCTD21*	cg04817034	Other	1.5E-02	−1.6
*LDB1*	cg07130508	TSS1500	4.3E-02	5.1
*MACF1*	cg22174844	Body	4.1E-02	−0.9
*ORM1*	cg13744452	Body	3.3E-07	−13.4
*ORM2*	cg13744452	Other	6.6E-08	−15.1
*SLC19A2*	cg08729004	Other	8.2E-03	3.0
*SPAG4*	cg26466505	Other	4.3E-02	3.3
*TALDO1*	cg13749939	Other	1.5E-02	1.5
*TBX5*	cg16559598	TSS1500	4.3E-02	−0.3
*U2AF1*	cg05809947	Other	2.9E-07	−2.2
*USP35*	cg04817034	Body	1.4E-02	−4.0
*ZNF259*	cg20691428	Other	2.0E-02	−1.2

Gene region reflects where the CpG is located relative to the gene, *P*-value reflects the strength of the correlation between methylation level and expression, and the coefficient reflects the direction of the correlation between methylation level and expression.

To validate the prognostic value of the discovered signature, the signature was applied to a validation set of breast cancer patients collected by TCGA (n = 583). The patients were divided into two groups with significantly different prognosis (HR = 2.31, *P* = 6.23e-4; Figure 4B).

The prognostic signature derived from patients with IBC was then applied to patients with DCIS and mixed DCIS-IBC tumors (n = 52). The patients were separated into groups with significantly different prognosis (*P* = 3.69e-2; Figure 4C). The good prognosis group included 14 pure DCIS and 15 mixed DCIS-IBC, while the bad prognosis group included 7 pure DCIS and 16 mixed DCIS-IBC. Comparing prognosis in DCIS versus mixed DCIS-IBC showed that the patients with mixed lesions had significantly adverse prognosis. In fact, only one breast cancer-specific death was observed among the patients with pure DCIS.

Multivariable Cox proportional hazard models were calculated for the patients in the training set (n = 176) as well as patients in the TCGA validation (n = 583) adjusting for estrogen receptor (ER) status, *TP53* mutation status (only training set), T status, and lymph node status. Classification by the prognostic signature was significantly associated with survival in both data sets (*P* < 0.001 and *P* = 0.008, respectively; Table 5). In addition to the prognostic signature, lymph node status was significantly associated with survival in the TCGA validation set. Importantly, combining lymph node status and classification by the prognostic signature provided an even better segregation of patients (*P* = 8.26e-5; Figure 4D). Patients that were both lymph node-negative and had a low index had the best prognosis, while patients that were lymph node-positive and had a high index had the worst prognosis. PAM50 classification was not significant in either of the patient cohorts.

**Table 5:** **Multivariate Cox proportional hazard analyses**

	**HR**	**HR 95% confidence interval**	***P*** **-value**
**Training set**			
ER status	1.32	0.62-2.84	0.470
*TP53* mutation	1.08	0.48-2.45	0.850
T status (2)	2.14	0.89-5.13	0.088
T status (3 or 4)	1.48	0.47-4.59	0.502
Lymph node status	2.22	0.89-5.51	0.087
Prognostic signature	29.0	9.36-89.9	5.4E-09
**TCGA validation**			
ER status	0.87	0.49-1.55	0.639
T status (2)	0.92	0.47-1.82	0.814
T status (3 or 4)	1.14	0.51-2.54	0.749
Lymph node status	2.13	1.14-3.98	0.018
Prognostic signature	2.09	1.21-3.59	0.008

## Discussion

Here we report the DNA methylation profiles of a breast cancer progression series, including normal breast tissue, DCIS, IBC and mixed lesions. Interestingly, most of the aberrations in the epigenetic profile were observed already in the pre-invasive DCIS stage. The affected pathways suggested that many of the changes may not occur in the tumor, but in infiltrating cells or at least in genes that enable cross-talk to such cells. Also of interest was that DNA methylation profiles of the basal type of breast cancer were more similar to normal tissue than were the luminal-like tumors. These data suggest that the methylation profiles may be a function of the cell of origin as much as a marker of progression. We also report a signature comprising DNA methylation levels of 18 CpGs that was prognostic for breast cancer patients with invasive tumors as well as for patients with DCIS and mixed lesions of DCIS and IBC. The signature was discovered in a training data set of 176 patients and validated in 583 patients from the TCGA. In the validation patient group the prognostic signature and lymph node status were complementary, potentially providing valuable information for clinical decision-making. The patients that were classified with good prognosis by DNA methylation and additionally were lymph node-negative might benefit from reduced or no adjuvant treatment, while patients that were classified with adverse prognosis by DNA methylation and were lymph node-positive could potentially benefit from more aggressive treatment.

A great advantage of DNA methylation is that it is relatively easy to design an assay that may be used in a clinical setting. DNA methylation can be measured on an absolute scale (from 0 to 100%), is stable in the cell over time, and is relatively insensitive to handling in the laboratory. This work clearly shows the potential of DNA methylation-based signatures for clinical utilization.

With data from two independent cohorts of normal tissue and DCIS, we report that the DNA methylation profiles of DCIS were radically changed compared with normal breast tissue, involving more than 5,000 genes. One cohort consisted of fresh frozen tissue and normal controls from healthy women, while the other cohort consisted of formalin-fixed paraffin-embedded (FFPE) DCIS samples and matched adjacent normal breast tissue. Thus, the reported changes in methylation levels across these genes appear to be independent of tissue preparation and the normal tissue’s proximity to tumor tissue. Comparably, the changes between DCIS and IBC were more modest, involving around 1,000 genes. These findings suggest that the epigenome is severely altered in the early neoplastic setting in the breast. Previous studies of breast cancer progression have also reported early aberrant DNA methylation in DCIS, but they characterized fewer genes (summarized in [11]). The current study has the advantage of a high coverage methylation assay (Illumina HumanMethylation450) and leverages true normal controls from healthy women. Our observation that extensive epigenetic alterations occur early in cancer progression has been reported for other cancer types, including colorectal cancer. For example, studies using the HumanMethylation450 assay reported that precancerous adenomas demonstrate heterogeneity similar to invasive tumors, and that aberrant DNA methylation occurs early in colorectal cancer formation [22].

Classification of breast cancer by hierarchical clustering showed that basal-like tumors clustered with the normal samples in one cluster, and luminal A and luminal B tumor clustered together in the second cluster (Figure 1). This observation largely recapitulates and extends the results from a previous study [8]. Since DNA methylation aberrations occur early in carcinogenesis, it is possible that DNA methylation changes may play a role in the development of molecular subtypes of breast cancer, although it is also possible that the correlation with methylation is a consequence of subtype. Future studies are needed to define the mechanistic effects that DNA methylation and other epigenetic marks may have on early development of cancer.

DCIS lesions tend to grow slower and show less inter-tumor heterogeneity than IBC lesions. Consequently, it would be pertinent to perform subtype-specific analyses of differences between DCIS and IBC. In the present study, however, the number of DCIS samples was too few to perform subtype-specific analyses. Future studies should aim to collect enough DCIS samples to divide both DCIS and IBC samples into intrinsic subtypes of breast cancer while including enough samples for statistical analyses. The inter-sample heterogeneity in the normal samples (mammoplastic reductions and needle biopsies from healthy women) was low compared with the neoplastic lesions (Figure 1).

Correlation between DNA methylation and gene expression was found throughout the genome and involved almost 3,000 genes. CpGs whose methylation level correlated with expression were enriched close to TSSs, but also found at distances up to 100 kb from them. Interestingly, about a quarter of the genes whose expression level correlated with methylation level showed a positive correlation, meaning that a higher methylation level was associated with higher expression. Viewed in relation to functional regions in genes, 70% of the positive correlations between methylation level and expression were found in the 3’ UTR or the gene body. Similar findings have been reported in chronic lymphocytic leukemia [25] and support that promoter hypermethylation is an important mechanism for gene silencing, while DNA methylation elsewhere may have more complex functions that are yet to be fully understood. Possible mechanisms for regulation of gene expression by non-promoter methylation include interplay between nucleosome positioning and chromatin structure, regulation of enhancer region availability, and/or gene body regulation of alternative promoters [25,26]. Statistical significance of correlation between DNA methylation and gene expression was corrected for multiple testing by Bonferroni correction. This method is very strict, and may underestimate the association between DNA methylation and gene expression.

The survival signature segregated patients with DCIS and mixed DCIS-IBC into two groups with significantly different prognosis. The signature classified most of the patients with mixed DCIS-IBC that experienced breast cancer-specific death into the bad prognosis group. Additionally, the single patient with pure DCIS that experienced breast cancer-specific death was also classified into the bad prognosis group. Since only one of the patients with pure DCIS died of breast cancer, it was not possible to perform the analysis on only patients with pure DCIS. Taken together, the signature may have great potential to classify patients with DCIS or mixed lesions according to prognosis, but more patients must be studied to further validate the clinical value.

Several of the genes in the survival signature have roles in tumor suppressive functions. The protein product of *IRF6* has been shown to function synergistically with the tumor suppressor maspin to regulate mammary epithelial differentiation [27], and has also been shown to have tumor suppressor activity in squamous cell carcinoma [28]. *TBX5* is a transcription factor that has been implicated as a tumor suppressor in colon cancer and has been found silenced by DNA methylation [29]. A SNP (rs1265507) located between *TBX5* and *TBX3* was also associated with mammographic density in a genome-wide association study [30]. In the present study, high methylation levels of CpGs in *TBX5* were associated with lower expression levels of *TBX5* and adverse prognosis. *DIEXF* is thought to be involved in the turnover of p53 [31], and *CEND1* has been shown to affect cyclin D1 levels [32]. *ZNF259* has been shown to be involved in regulation of the cell cycle through interactions with the epidermal growth factor receptor [33], and *KCTD21* is thought to act as a tumor suppressor in medulloblastoma by modulating Hedgehog signaling through degradation of histone deacetylase 1 [34].

Some genes in the survival signature have also been associated with functions related to motility and invasion: *EPN3* over-expression has been shown to promote cancer cell invasion [35], *MACF1* has been shown to be involved in cell mobility and steering by interactions with HER2 [36], and *CSNK1G2* is thought to modulate the activity of metastasis-associated *MTA1* while itself a target of ER [37]. Taken together, many of the genes associated with the survival signature have tumor suppressive functions or are involved in regulation of motility and ability to invade.

A strong immune component in breast tumors observed by measuring DNA methylation has previously been reported [38]. The genes that were differentially methylated between DCIS and IBC were borderline significantly enriched in the agranulocyte and granulocyte adhesion and diapedesis pathways, suggesting that many of the observed changes may occur in infiltrating cells or in genes that enable cross-talk to such cells.

*CUL7* (cullin 7) methylation levels increased from both normal to DCIS and DCIS to IBC. *CUL7* encodes a ubiquitin ligase that forms complexes with p53 and Parc. It was shown to regulate apoptosis independently of p53 [39]. In another report [40], *CUL7* was shown to function as an antiapoptotic oncogene through cooperation with Myc in a p53-dependent manner. Also, *CUL7* has been shown to be involved in liver carcinogenesis [41]. Importantly, *CUL7* has not previously been reported in breast cancer. *ICAM2* (Intercellular adhesion molecule 2) methylation levels decreased between normal and DCIS, and increased between DCIS and IBC. *ICAM2* is involved in cell adhesion and thought to play a role in immune response. In pancreatic cancer, *ICAM2* has been reported to have tumor suppressor function through immune surveillance [42].

## Conclusion

DNA methylation profiles of DCIS were radically changed compared with normal breast tissue while the changes between DCIS and IBC were comparably modest. A DNA methylation-based prognostic signature was reported that has potential in patients both with invasive breast cancer and with *in situ* carcinoma. Correlation between DNA methylation and gene expression was observed in a substantial part of the genome, and both positive and negative correlations were observed.

## Materials and methods

### Patient material

Material for this study was obtained from 285 fresh frozen tissue samples representing different progression stages of breast cancer; 46 normal samples, 22 pure DCIS, 31 mixed DCIS-IBC and 186 pure IBC were included. Of the 46 normal samples, 17 were tissue from mammoplastic reductions of healthy women collected at Akershus University Hospital (institutional review board (IRB) approval number 429–04148). Twenty-nine needle biopsies from healthy women and 49 IBC samples were collected at the Norwegian Radium Hospital (IRB approval number S-02036) [43]. DCIS samples, mixed DCIS-IBC samples, and 15 of the IBC samples were collected at Uppsala Academic Hospital (IRB approval number Dnr 2005:118) [44,45]. Of the pure DCIS samples, 18 of 22 had a tumor component of >75% [44]. The 123 IBC samples were collected from hospitals in the Oslo region (IRB approval number S-97103) [46]. The IBC samples were predominantly stage I and II. All studies are in compliance with the Helsinki Declaration and were approved by local ethical committees and local authorities.

### DNA methylation analysis

The DNA methylation status of more than 450,000 CpG sites was interrogated using Illumina Infinium HumanMethylation450 microarray. The returned value of each CpG probe is called β and is calculated as the methylated signal divided by the sum of the methylated and the unmethylated signal. β thus represents the fraction of methylated DNA molecules at a specific locus.

### Preprocessing of DNA methylation data

Preprocessing and normalization involved steps of probe filtering, color bias correction, background subtraction and subset quantile normalization as previously described [47]. After preprocessing of the data, 468,424 CpG probes were included. The normalized data as well as the raw data are available in Gene Expression Omnibus (GEO) with accession number GSE60185.

### Gene expression analysis

mRNA expression data were available for a subset of 104 of the IBC samples studied here. An Agilent whole genome 4x44K oligo array was used for the mRNA analysis as previously described [48]. The mRNA expression data are available in GEO with accession number GSE19783. Molecular subtypes of breast cancer (luminal A, luminal B, HER2-enriched, basal-like and normal-like) were determined using the PAM50 gene list.

### Methylation data processing

Statistical and bioinformatical analyses of the methylation level of the 285 samples were performed on two individual datasets, one including methylation levels of all 468,424 CpGs, and one including only 'gene region collapsed' data. The 'gene region collapsed' methylation data were constructed to reduce the dimensions of the methylation data and to focus the analysis on regions that are most relevant for gene function. A CpG that is mapped to a gene is located in one of six subregions: TSS1500, TSS200, 5’ UTR, first exon, body and 3’ UTR. These subregions represent: 1) CpGs that are between 1,500 and 200 bp upstream of the TSS; 2) CpGs that are between 200 bp upstream of the TSS and the TSS itself; 3) CpGs in the 5’ UTR; 4) CpGs in the first exon; 5) CpGs in other exons or in introns (body); and 6) CpGs in the 3’ UTR. Methylation levels for each subregion were summarized using the median. In this approach intergenic CpGs will not be included. The resulting gene region collapsed dataset had 88,909 targets.

### Methylation changes during progression of breast cancer

SAM was used to identify differentially methylated loci between normal and DCIS, and between DCIS and IBC. The analysis was performed using the *SAM* function of the R package *samr* [49] with 100 permutations. For a locus to be considered differentially methylated, the difference between the median methylation levels in the two groups had to be at least 0.1 (10%) and the FDR q-value had to be smaller than 0.01 (1%).

### Hierarchical clustering

Hierarchical clustering was performed using the methylation level of the 500 most variable gene regions. The distance matrix was calculated using Pearson correlation and average linkage was applied.

### Correlation between DNA methylation and gene expression

Correlation between DNA methylation level and gene expression was investigated by two approaches. First, if a CpG was within 100 kb of the TSS of a gene, the methylation level of the CpG and expression of the gene were tested for non-zero correlation using Pearson correlation (*eMap1* function in the R package *eMap*) [50]. For both analyses an association was considered significant if the Bonferroni corrected *P*-value was smaller than 0.05. Genome-wide correlation between methylation and expression was visualized using the R package *quantsmooth* [51]. Second, the median methylation level of CpGs in the 'gene region collapsed' data and gene expression of the corresponding gene was tested for non-zero correlation using Pearson correlation (R function *corr.test*).

### Survival analysis

Lasso regularization [52,53] was applied to identify CpGs for which methylation level predicted survival (*cv.glmnet* function in the R package *glmnet*) [54]. This approach gives a signature of targets that together capture the variation that is associated with survival of patients. Pre-selection was performed before regression in order to reduce the number of possible CpG sites and to focus on the ones correlated with expression. Univariate absolute correlation between methylation level and expression with nominal *P*-value lower than 0.05 were used to preselect data, and 182,653 CpGs were included in the analysis. The analysis was run 100 times and the probes that were present in 80% of the resulting lists were used in the final signature. The coefficients were calculated as the mean of the coefficients in all lists where the probe was present. Patients were divided into high- and low-risk groups according to the following index for patient i:

$$ inde{x}_i={\displaystyle \sum_{g=1}^n{\beta}_g}\cdot {X}_{g_i} $$

where g is the target (CpG or gene), n is the number of targets, β_g_ is the Lasso coefficient for target g and X_gi_ is the methylation value for target g in patient i. Kaplan-Meier estimator and log-rank tests were performed using the functions *Surv*, *survfit* and *survdiff* (R package *survival*) [55]. Breast cancer-specific survival was used in all analyses.

Multivariate Cox proportional hazard survival analysis was performed using the function *coxph* (R package *survival*) to adjust for ER status, *TP53* mutation status, T status and lymph node status. Each parameter in the multivariate model was investigated for violations of the assumption of proportional hazards using the function *cox.zph* (R package *survival*).

### Validation cohort of adjacent normal tissue and DCIS

To validate the methylation changes between normal tissue and DCIS, an independent set of DCIS and adjacent normal tissues was profiled using the Illumina Infinium HumanMethylation450 array. FFPE pure DCIS (n = 40) and adjacent normal tissue (n = 15) underwent pathology review and 2 mm core punches were taken for processing as described in the Illumina Infinium FFPE Restoration solution protocol. The methylation data were preprocessed using the R package ChAMP [56] and 397,600 probes out of 485,577 remained after quality control. A gene region collapsed data set was also constructed for this data set as described above.

### Validation cohort from The Cancer Genome Atlas

To validate the prognostic signature, DNA methylation data and clinical information were downloaded from TCGA data portal [9]. Only breast cancer patients for whom there were both overall survival data and tumor DNA methylation analysis had been performed by Illumina HumanMethylation450 were used for validation (n = 583). Probes with more than 50% missing values were removed, and further missing values were imputed using the function *pamr.knnimpute* (R package *pamr*) [57] with k = 10.

### Data analysis

All analyses were performed using the R computing framework [58]. Gene lists were analyzed with Ingenuity Pathways Analysis (Ingenuity® Systems, Redwood, California, USA).

## Additional files

Additional file 1:
**Significance Analysis of Microarrays (SAM) analysis of methylation level of gene regions between the five PAM50 derived subtypes.** Gene region, false discovery rate (FDR) q-value and median methylation for each PAM50 subtype. Additional file 2:
**Hierarchical clustering and heatmap of invasive tumors using gene regions differentially methylated between the five gene expression-derived subtypes.**
Additional file 3:
**Correlation between DNA methylation and gene expression; individual CpGs.** Illumina probe ID, gene, transcription start site (TSS), chromosome, position of CpG, CpG position relative to CpG islands (CGIs) and gene region, correlation coefficient and uncorrected *P*-value. Additional file 4:
**Correlation between DNA methylation and gene expression; functional gene regions.** Gene, gene region (whose methylation level correlates with expression), correlation coefficient and uncorrected *P*-value. Additional file 5:
**SAM analysis of methylation level of individual CpGs between healthy breast tissue and DCIS.** Illumina probe ID, gene, CpG position relative to CGIs and gene region. Analyses performed in two independent datasets. Only concordant results in both datasets are reported. Additional file 6:
**SAM analysis of methylation level of individual CpGs between DCIS and IBC.** Illumina probe ID, gene, CpG position relative to CGIs and gene region. Additional file 7:
**SAM analysis of methylation level of gene regions between healthy breast tissue and DCIS.** Analyses performed in two independent datasets. Only concordant results in both datasets are reported. Additional file 8:
**SAM analysis of methylation level of gene regions between DCIS and IBC.**

## Abbreviations

bp: base pair
CGI: CpG island
DCIS: ductal carcinoma *in situ*
ER: estrogen receptor
FDR: false discovery rate
FFPE: formalin-fixed paraffin-embedded
GEO: Gene Expression Omnibus
HR: hazards ratio
IBC: invasive breast carcinoma
IRB: institutional review board
SAM: Significance Analysis of Microarrays
TCGA: The Cancer Genome Atlas
TSS: transcription start site
UTR: untranslated region
