# Supplementary figures and images for: Genome-wide DNA methylation profiles in progression to in situ and invasive carcinoma of the breast with impact on gene transcription and prognosis
Source: Genome Biol. 2014 Aug 22;15(8):435. doi: 10.1186/s13059-014-0435-x (PMC4165906; doi:10.1186/s13059-014-0435-x)

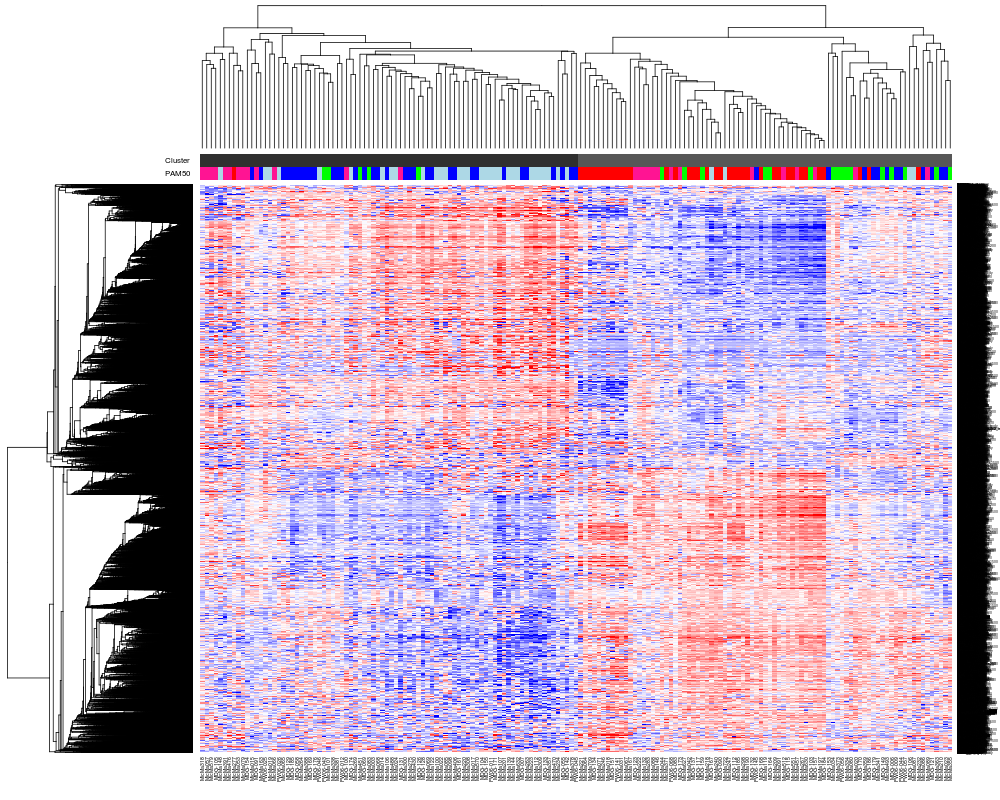

Supplement: Additional file 2: — Hierarchical clustering and heatmap of invasive tumors using gene regions differentially methylated between the five gene expression-derived subtypes. [file 13059_2014_435_MOESM2_ESM.png]
